# Retracted: The Effects of Cytokines in Adipose Stem Cell-Conditioned Medium on the Migration and Proliferation of Skin Fibroblasts In Vitro

**DOI:** 10.1155/2020/4587608

**Published:** 2020-09-28

**Authors:** BioMed Research International

BioMed Research International has retracted the article titled “The Effects of Cytokines in Adipose Stem Cell-Conditioned Medium on the Migration and Proliferation of Skin Fibroblasts In Vitro” [1]. The article is one of four other articles [2–5] published by the same group of authors in 2012 and 2013. It was found to contain duplicated figures and data, where some of the cytokine expression data presented in Table 1 of [1] are repeated in Table 3 of [2], Table 1 of [4], and Table 1 of [5]. Details of the figure duplication are as follows:

a. Figure 1d in [1] is the same as Figure 2 (a1 - a4) in [2] and Figure 2E in [3].

b. Figure 2a in [1] is the same as Figure 6a in [2] and Figure 2H in [3], but rotated.

The authors apologize for these errors and agree to the retraction of the article. The author's institution investigated our concerns and concluded that these errors were not the result of academic misconduct. The article is being retracted with the agreement of the journal and the editorial board due to concerns regarding the reliability of the data.
